# A Retrospective Analysis of Patients with Short Stature in Eastern China between 2013 and 2019

**DOI:** 10.1155/2021/6640026

**Published:** 2021-04-20

**Authors:** Qianqian Zhao, Mei Zhang, Yuntian Chu, Hailing Sun, Hui Pan, Bo Ban

**Affiliations:** ^1^Department of Endocrinology, Affiliated Hospital of Jining Medical University, Jining Medical University, 89 Guhuai Road, Jining, Shandong 272029, China; ^2^Department of Endocrinology, Qingdao University, Qingdao 266000, China; ^3^Chinese Research Center for Behavior Medicine in Growth and Development, 89 Guhuai Road, Jining, Shandong 272029, China; ^4^School of Health Management and Medicine, Tongji Medical College, Huazhong University of Science and Technology, Wuhan, Hubei 430030, China; ^5^Key Laboratory of Endocrinology of National Health and Family Planning Commission, Department of Endocrinology, Peking Union Medical College Hospital, Chinese Academy of Medical Science and Peking Union Medical College, Beijing 100730, China

## Abstract

**Objective:**

To identify the aetiology of growth and development diseases and assess the long-term effectiveness of recombinant human growth hormone (rhGH) therapy in a real-life clinical setting and provide better guidance in clinical strategy and decision making.

**Methods:**

This retrospective study included 1145 children and adolescents with short stature admitted to the Department of Endocrinology, Affiliated Hospital of Jining Medical University, from January 2013 to December 2019, of whom 484 received rhGH treatment. The related anthropometrics and laboratory examinations were assessed in all participants.

**Results:**

A total of 1145 children and adolescents with short stature aged 10.5 ± 3.3 years, including 740 boys and 405 girls, were analysed in this study. The number of children and adolescents with short stature gradually increased per year from 2013 to 2019. The mean pretreatment height standard deviation score (SDS) and insulin-like growth factor-1 SDS were −2.93 ± 1.05 and -1.01 (-1.83--0.16), respectively. The majority of the children (658, 57.47%) were prepubescent. In total, 484 subjects aged 10.6 ± 3.2 years received rhGH and were followed up, and among them, 292 children were treated for more than one year. As the treatment time increased, the children's height SDS gradually increased, and most of them attained a height SDS within the normal range. The mean height SDS in children who were treated for more than one year was −3.0 ± 1.0 at baseline and gradually increased to −0.8 ± 0.3 by year 6. The results were consistent across subgroups of different aetiologies of short stature.

**Conclusions:**

Increasing attention has been given to the height of children during the period of 2013–2019 in eastern China. The present findings indicate that children with short stature need to be referred to a specialist centre to diagnose the cause of growth failure and that short children receiving rhGH therapy show a significant increase in height over time.

## 1. Introduction

Short stature refers to individuals in a similar living environment and of the same race, same sex, and similar age who are 2 standard deviations lower than the average height of the normal population [1]. An epidemiological study described that the incidence of primary and middle school students with short stature in China is approximately 3.16% [2]. Short stature is the result of multiple aetiologies, which are classified as pathological and normal variation. Pathological variants include growth hormone deficiency (GHD), hypothyroidism, idiopathic short stature (ISS), and Turner syndrome, whereas physiological variants include familial short stature (FSS), constitutional delay of growth and puberty (CDGP), and small for gestational age (SGA) [3, 4]. The physical and psychological development of patients with short stature is affected, the risk of cardiovascular disease increases significantly [5], and the health-related quality of life score is significantly reduced [6]. Children with short stature suffer from impaired self-esteem and social ability [7]. Recombinant human growth hormone (rhGH) therapy was approved for growth disorders [8]. rhGH replacement therapy in children with short stature can not only improve height [9] but also improve both blood lipid metabolism [10, 11] and quality of life [12]. It is widely known that there are regional and population differences in short stature, and previous studies have described the clinical data of children and evaluated children with short stature and poor growth during the period of 2007-2015 in southern China [13]. However, the data description of children with short stature in eastern China is limited.

This study describes the clinical data of children admitted to the Department of Endocrinology, Affiliated Hospital of Jining Medical University, for the evaluation of short stature and poor growth during the period of 2013–2019 in eastern China. This study is aimed at identifying the aetiology of growth and development diseases and assessing the long-term effectiveness of rhGH therapy in a real-life clinical setting and providing better guidance for clinical strategy and decision making. Growth and Development Diseases in Shandong Province: a cohort follow-up (GDDSD) study was specifically designed by the Affiliated Hospital of Jining Medical University to collect enrolment demographics as well as clinical and laboratory data from children and adolescents who were referred to the hospital for growth failure or were treated with rhGH.

## 2. Subjects and Methods

### 2.1. Subjects

The GDDSD study is an ongoing prospective, observational, open cohort study to evaluate the aetiology of growth and development diseases and the long-term safety and effectiveness of growth hormone therapy in a real-life clinical setting. All subjects were enrolled from the GDDSD study (http://www.chictr.org.cn, ChiCTR1900026510). A total of 1145 children and adolescents with short stature (740 males and 405 females) from January 2013 to December 2019 at the Department of Endocrinology, Affiliated Hospital of Jining Medical University, were enrolled. Among them, 484 subjects received rhGH and were followed up, and all patients receiving rhGH treatment met the exclusion contraindications, such as intracranial tumours. All the subjects who were short stature, which refers to the height of an individual, of more than two standard deviations (SD) below the corresponding mean height for a given age, sex, and ethnic group were included [14]. The exclusion criteria were as follows: children without a final diagnosis, clinical data for children and adolescents missing, and short stature with precocious puberty.

The study was approved by the Human Ethics Committee of the Affiliated Hospital of Jining Medical University (Shandong, China). All of the families of the patients were informed of the aims of the study, and a specific written informed consent form, from a standard source, was signed by all of the participants' parents.

### 2.2. Anthropometric Measurements

Height and weight were assessed following standard procedures. Height was expressed as the SDS based on normative values for Chinese children [15]. Body mass index (BMI) was calculated as the weight divided by the height in metres squared and expressed as the SDS according to the Chinese children and adolescent growth charts [16]. Pubertal status was evaluated by physical examination, and boys with no pubic hair and a testicular volume less than 4 ml or girls with no pubic hair and no breast development were considered prepubescent [17].

### 2.3. Laboratory Measurements

Two stimulating tests were performed to assess growth hormone (GH) secretion (the first one, 500 mg of levodopa for those weighing more than 30 kg; 250 mg of levodopa for those weighing less than 30 kg, orally; and the second one, 0.1 U/kg insulin, subcutaneously). GH concentration measurements were performed at 0, 30, 60, 90, and 120 min after administration. A chemiluminescence method was used to assess the GH concentration (ACCESS2, Beckman Coulter; USA), and the sensitivity was 0.010 *μ*g/l. Insulin-like growth factor-1 (IGF-1) and IGF-binding protein-3 (IGFBP-3) were measured by a chemiluminescence assay (DPC IMMULITE 1000 analyser, SIEMENS, Germany) with intra- and interassay CVs of 3.0% and 6.2% for IGF-1 and 4.4% and 6.6% for IGFBP-3, respectively. A GH peak in the GH stimulation test of less than 10 ng/ml was defined as GHD [18]. The IGF-1 SDS for age and sex was calculated according to IGF-1 levels for healthy children and adolescents of the same age and sex [19].

### 2.4. X-Ray Bone Age (BA) Assessment

BA was assessed using a radiograph of the left hand and wrist (Ysio, SIEMENS, and Germany). All radiographs were analysed by an experienced radiologist blinded to the patients' chronological ages using the Greulich and Pyle method [20].

### 2.5. Statistical Analysis

Statistical analysis was performed with R 3.4.3 (https://www.R-project.org) and EmpowerStats (https://www.empowerstats.com, X&Y Solutions, Inc., Boston, MA). Normally distributed variables are expressed as the mean ± standard deviation (SD), abnormally distributed variables are shown as the median (quartile), and categorical variables are expressed as frequencies or percentages.

## 3. Results

### 3.1. Aetiology Classification

The aetiology classification and rhGH treatment with short stature are shown in Table 1. Among the 1145 cases of short stature, there were 730 cases of short stature with disease, 579 of which were GHD, 8 cases were multiple pituitary hormone deficiencies (MPHD), 46 cases were hypothyroidism, 40 cases were SGA, 12 cases were skeletal development disorder, 12 cases were intracranial tumour, 16 cases were chromosomal disease, and 17 cases were chronic systemic disease. There were 415 cases of short stature without disease, 276 of which were ISS, 122 cases were FSS, and 17 cases were physical puberty delay. Of the 1145 patients, 484 received GH treatment, including 316 boys and 168 girls.

### 3.2. Clinical Characteristics

**Figure 1:**
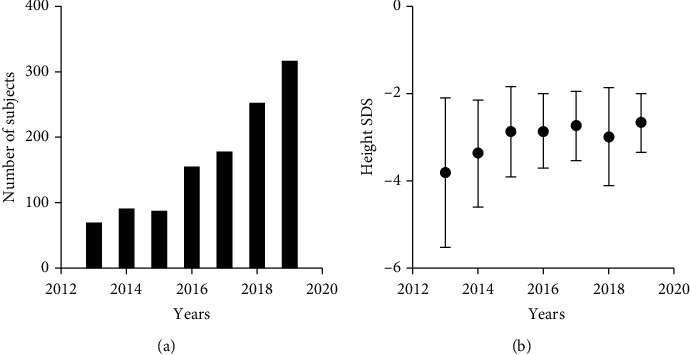
Number (a) and height SDS (b) of patients for the entire group from 2013 to 2019.

The clinical and biochemical characteristics of the children and adolescents with short stature are summarized in Table 2. A total of 1145 children and adolescents with short stature aged 10.5 ± 3.3 years, including 740 boys and 405 girls, were enrolled in this study. The mean bone age of the subjects was 9.4 ± 3.7 years. The mean pretreatment height SDS and IGF-1 SDS were −2.93 ± 1.05 and -1.01 (-1.83--0.16), respectively. The majority of the children, 658 (57.47%), were prepubescent. As shown in Table 2 and Figure 1, the number and height of the children and adolescents who were referred to the hospital increased.

### 3.3. Treatment Efficacy

**Figure 2:**
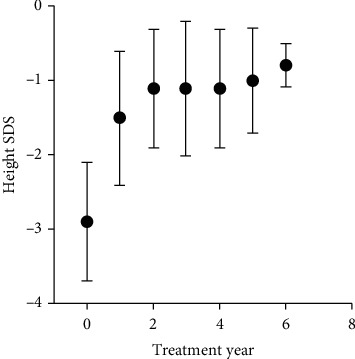
Height SDS by year of treatment (mean ± SD) for treated children.

As shown in Table 3, 484 subjects aged 10.6 ± 3.2 years received rhGH and were followed up. The median duration of rhGH treatment was 1.00 (1.00-2.00) years, and the number of treated children decreased with time. Among them, 192 children have been treated for less than one year, and the remaining 292 children have been treated for more than one year. As shown in Figure 2, as the treatment time increases, the children's height SDS gradually increases, and most of them will have a height SDS within the normal range. The mean height SDS in children treated for more than one year was −3.0 ± 1.0 at baseline and gradually increased to −0.8 ± 0.3 by year 6 (Table 4). In addition, from Figure 2 and Table 4, we observe that the treatment effect is very significant after 1 year of rhGH treatment, and the height SDS increases significantly (*P* < 0.001).

In addition, we described height improvement in children treated with rhGH for more than one year according to different aetiologies of short stature (e.g., GHD, ISS, and FSS). We observed that the increase in height over time was consistent with the general population in each subgroup (Tables S1-S5). Among the 292 children who received rhGH therapy for at least 1 year, the number with MPHD (*n* = 2), chromosomal disease (*n* = 2), chronic systemic disease (*n* = 1), skeletal development disorder (*n* = 1), or physical puberty delay (*n* = 1) was relatively small, and this study did not include them in the analysis.

## 4. Discussion

Our study describes the general situation and treatment of short stature in China in the past 7 years. Our findings showed that the causes of short stature are complex and varied, and short stature in children and adolescents may be due to changes in normal growth or pathological conditions. In eastern China, the number of short patients whose parents sought medical attention and who were referred to endocrinologists is increasing every year. Furthermore, with rhGH therapy, which has been approved for patients with short stature, increases in the mean height SDS were observed with increasing treatment time, and most of the patients reached the normal range of height.

Our previous study found that the prevalence rate of short stature was 3.16% [21], which was consistent with the findings of Wang et al., who investigated the epidemiology of short stature among primary and middle school students in Anhui Province, China [2]. Short stature may result from various causes, which may be primary or secondary growth disorders. Accurate evaluation and monitoring of children's growth is essential for the early detection of defects related to treatable conditions and early identification of growth variations related to normal conditions [22]. The aetiology of short stature in the population of this study was similar to that reported in northern China [13]. However, our study subjects were all children and adolescents eligible for short stature, and the previous study population included children who could not be diagnosed with short stature because their average height SDS was −2.37 ± 1.05, which means that the height of some children was not less than -2 SDS. In addition, we observed that the proportion of short children with disease was higher than the proportion of children with short stature without disease. However, a previous study [23] reported that the majority of children with short stature are normal variants, and the reason for the differences between this study and other studies is unclear. However, there may be biases in referrals to tertiary hospitals or ethnic differences in regional populations. Furthermore, we found that GHD and ISS were the most common causes of short stature, which is consistent with previous findings [24].

The prevalence of short stature has increased significantly in the past 7 years. Sex differences are more obvious in children with short stature in the hospital. There are obviously more boys than girls, which is consistent with previous study reports [13]. Furthermore, in many countries, rhGH treatment of children with short stature appears to be sex-biased; boys are more likely to receive rhGH treatment regardless of the underlying diagnosis of the cause of short stature [25, 26]. In our study, this difference is also significant, and it may be related to the patients, social concepts, and family factors. In terms of height, boys have more social pressure than girls, including employment and marriage that increases the number of boys who seek treatment from specialists, and this phenomenon is understandable in China.

In our study, there was a large proportion of prepubescent children, suggesting that age plays an important role in the evaluation of patients with short stature. Early evaluation, diagnosis, and treatment are beneficial for height improvement. Polak et al. found that compared with older age at the beginning of treatment, younger age at the beginning of treatment is related to improvement in height [27]. Furthermore, a previous study has shown that the height before puberty in patients with short stature is significantly related to the final adult height [28]. However, if a patient with short stature cannot be diagnosed early, delaying the timing of treatment may result in the loss of the opportunity to improve height. In our study, all children with short stature were admitted to the hospital at a relatively late age, which increased the difficulty of treatment. Therefore, we need to provide more education regarding short stature and increase awareness of the condition and early treatment.

Among the 1145 cases in this study, 484 children with short stature received rhGH therapy, with a treatment rate of 42.3%, which is similar to previous reports [29, 30]. It is well known that rhGH treatment has a good growth-promoting effect on children with short stature [8]. With the increase in treatment time, the height SDS of children with short stature is increasing year by year. Moreover, in our study, during the first year of treatment, the acceleration was slightly better, and the response was very similar for the following 6 years. This finding was consistent with previous studies [31, 32]; a study conducted in 200 children treated for ISS showed that rhGH treatment can increase the height of children with ISS, with the highest growth rate in the first year and the growth rate in the second year being significantly lower than that in the first year [31]. In addition, this finding is similar to the findings of Kemp et al., who analysed 47,226 patients in the National Cooperative Growth Study (NCGS) and observed better height growth during the first year of therapy [32]. These findings suggest that it is essential to evaluate the aetiology and provide early treatment for children with short stature.

The present study has several limitations. First, this study is a retrospective study. In this study, we only described the aetiology and general characteristics of children with short stature but did not discuss the related relationship in depth. Second, we failed to study the underlying causes of the sex differences in the treatment of short children. Finally, in this study, the factors related to the efficacy of rhGH therapy were not explored and deserve further study.

In conclusion, increasing attention has been given to the height of children during the period of 2013–2019 in eastern China. The present findings indicate that children with short stature need to be referred to a specialist centre to diagnose the cause of growth failure. Early treatment with rhGH is recommended since children with short stature respond well to rhGH therapy. In addition, studies on the influencing factors of height growth and follow-up to adult lifetime height are directions for further research in the future.

## Figures and Tables

**Table 1:** Aetiology classification and rhGH treatment with short stature.

Aetiology classification	Cases		rhGH treatment	
		Boy/girl		Boy/girl
*Short with disease*	730	464/266	334	216/118
GHD	579	374/205	271	178/93
Complete GHD	219	145/74	111	77/34
Partial GHD	360	229/131	160	101/59
MPHD	8	6/2	6	4/2
Hypothyroidism	46	24/22	26	15/11
SGA	40	29/11	15	9/6
Skeletal development disorder	12	5/7	3	2/1
Intracranial tumour	12	10/2	0	0
Chromosomal disease	16	2/14	6	1/5
Turner syndrome	14	0/14	5	0/5
Other chromosomal disease	2	2/0	1	1/0
Chronic systemic disease	17	14/3	7	7/0
*Short without disease*	415	276/139	150	100/50
ISS	276	174/102	85	53/32
FSS	122	88/34	63	45/18
Physical puberty delay	17	14/3	2	2/0
Total	1145	740/405	484	316/168

Abbreviations: rhGH: recombinant human growth hormone; GHD: growth hormone deficiency; MPHD: multiple pituitary hormone deficiency; SGA: small for gestational age; ISS: idiopathic short stature; FSS: familial short stature.

**Table 2:** Baseline clinical and biochemical characteristics of the subjects.

Year	All	2013	2014	2015	2016	2017	2018	2019
Number	1145	68	90	86	155	178	252	316
Sex (male %)	740 (64.63%)	45 (66.18%)	59 (65.56%)	56 (65.12%)	108 (69.68%)	111 (62.36%)	150 (59.52%)	211 (66.77%)
Age (years)	10.5 ± 3.3	10.4 ± 3.3	10.6 ± 3.1	10.2 ± 3.1	9.5 ± 3.4	10.3 ± 3.2	10.3 ± 3.1	11.4 ± 3.5
Bone age (years)	9.4 ± 3.7	8.7 ± 3.5	9.2 ± 3.5	9.0 ± 3.9	8.3 ± 3.9	9.4 ± 3.6	9.9 ± 3.6	9.7 ± 3.6
Height (cm)	131.43 ± 18.04	127.70 ± 16.60	129.99 ± 15.54	130.02 ± 17.22	127.14 ± 18.96	131.69 ± 18.18	134.21 ± 17.43	132.76 ± 18.71
Height SDS	−2.93 ± 1.05	−3.80 ± 1.71	−3.36 ± 1.23	−2.87 ± 1.03	−2.85 ± 0.85	−2.74 ± 0.80	−2.66 ± 0.69	−2.98 ± 1.13
Weight (kg)	31.90 ± 13.22	28.62 ± 10.49	30.79 ± 10.41	29.65 ± 10.97	29.58 ± 11.67	31.80 ± 13.03	33.55 ± 13.76	33.43 ± 14.96
BMI (kg/m^2^)	17.58 ± 3.43	16.70 ± 2.67	17.74 ± 3.23	16.90 ± 2.74	17.47 ± 3.00	17.53 ± 3.28	17.86 ± 3.59	17.78 ± 3.88
BMI SDS	-0.15 (-0.91-0.78)	-0.38 (-1.45-0.39)	-0.45 (-0.96-0.33)	-0.33 (-1.18-0.60)	-0.10 (-0.79-0.83)	-0.16 (-0.86-0.95)	0.03 (-0.62-0.97)	-0.18 (-0.91-0.73)
Peak GH (ng/ml)	7.44 (4.83-11.49)	7.03 (5.68-10.20)	8.92 (4.07-12.81)	9.25 (5.77-14.80)	7.53 (4.86-12.21)	8.02 (4.77-11.96)	6.58 (4.45-9.57)	7.72 (5.11-10.79)
IGF-1 (ng/ml)	197.00 (114.00-319.00)	157.50 (83.03-218.01)	189.00 (98.10-279.00)	168.00 (109.25-279.50)	173.00 (90.55-284.00)	200.50 (125.75-296.50)	220.00 (139.00-360.00)	212.00 (126.00-345.23)
IGF-1 SDS	-1.01 (-1.83--0.16)	-1.25 (-1.96--0.47)	-1.35 (-2.36--0.34)	-1.23 (-2.08--0.34)	-1.08 (-1.89--0.22)	-0.96 (-1.79--0.18)	-0.77 (-1.58-0.04)	-1.02 (-1.70--0.12)
IGFBP-3 (*μ*g/ml)	4.86 ± 1.71	4.28 ± 1.24	4.61 ± 1.34	4.21 ± 1.27	4.92 ± 1.47	4.34 ± 1.22	5.13 ± 1.38	5.20 ± 2.30
Pubertal stage								
In prepuberty (%)	658 (57.47%)	36 (52.94%)	53 (58.89%)	55 (63.95%)	88 (56.77%)	106 (59.55%)	134 (53.17%)	186 (58.86%)
In puberty (%)	487 (35.37%)	32 (47.06%)	37 (41.11%)	31 (36.05%)	67 (43.23%)	72 (40.45%)	118 (46.83%)	130 (41.14%)

Abbreviations: height SDS: height standard deviation scores; BMI SDS: body mass index standard deviation scores; IGF-1 SDS: insulin-like growth factor-1 standard deviation scores; IGFBP-3: insulin-like growth factor-binding protein-3; peak GH: peak growth hormone; normal distribution of data was presented as mean ± standard deviation; nonnormal distribution of data was presented as median (interquartile range) and categorical data using number (percentage).

**Table 3:** Clinical and biochemical characteristics of the rhGH-treated subjects.

Year	All	2013	2014	2015	2016	2017	2018	2019
Number	484	20	33	38	76	82	75	160
Sex (male %)	316 (65.29%)	14 (70.00%)	19 (57.58%)	28 (73.68%)	51 (67.11%)	58 (70.73%)	45 (60.00%)	101 (63.12%)
Age (years)	10.6 ± 3.2	10.3 ± 3.3	10.1 ± 2.4	10.4 ± 3.3	9.7 ± 3.4	10.6 ± 3.1	10.4 ± 3.0	11.2 ± 3.2
Bone age (years)	9.5 ± 3.6	9.2 ± 3.5	8.9 ± 3.0	9.2 ± 4.2	8.4 ± 3.7	9.6 ± 3.5	9.8 ± 3.5	10.1 ± 3.5
Height (cm)	131.51 ± 18.06	127.44 ± 16.63	127.24 ± 12.56	129.47 ± 18.79	127.31 ± 18.82	132.86 ± 17.79	133.57 ± 17.14	133.73 ± 18.85
Height SDS	−2.88 ± 0.81	−3.69 ± 1.69	−3.54 ± 1.31	−2.99 ± 1.28	−2.81 ± 0.78	−2.74 ± 0.73	−2.70 ± 0.82	−2.80 ± 0.98
BMI (kg/m^2^)	17.70 ± 3.35	16.86 ± 2.52	17.51 ± 3.25	17.50 ± 3.05	17.66 ± 3.03	17.77 ± 3.46	17.20 ± 2.70	18.11 ± 3.85
Peak GH (ng/ml)	6.78 (4.34-9.88)	6.73 (5.66-7.85)	4.34 (2.92-9.24)	7.44 (5.20-10.94)	6.70 (4.52-10.91)	6.64 (4.13-11.21)	7.11 (4.73-9.51)	6.38 (4.79-9.98)
IGF-1 (ng/ml)	196.00 (125.25-316.75)	173.87 (48.97-217.19)	171.50 (122.00-218.75)	158.00 (109.75-293.25)	180.00 (95.00-240.00)	201.50 (128.25-355.00)	216.50 (154.00-349.00)	206.00 (137.00-358.00)
IGF-1 SDS	-1.02 (-1.77--0.19)	-1.15 (-1.67--0.64)	-1.38 (-2.30--1.02)	-1.37 (-2.30--0.10)	-1.08 (-1.79--0.28)	-1.00 (-1.79--0.19)	-1.07 (-1.71--0.22)	-0.58 (-1.35-0.26)
Duration of rhGH treatment (years)	1.00 (1.00-2.00)	2.00 (1.00-3.50)	2.00 (1.00-3.00)	1.00 (1.00-2.00)	1.00 (1.00-2.00)	1.00 (1.00-2.00)	1.00 (1.00-2.00)	1.00 (1.00-1.00)
Pubertal stage								
In prepuberty (%)	273 (56.40%)	11 (55.00%)	21 (63.64%)	25 (65.79%)	41 (53.95%)	47 (57.32%)	42 (56.00%)	86 (53.75%)
In puberty (%)	211 (43.60%)	9 (45.00%)	12 (36.36%)	13 (34.21%)	35 (46.05%)	35 (42.68%)	33 (44.00%)	74 (46.25%)

Abbreviations: height SDS: height standard deviation scores; IGF-1 SDS: insulin-like growth factor-1 standard deviation scores; peak GH: peak growth hormone; rhGH: recombinant human growth hormone; normal distribution of data was presented as mean ± standard deviation; nonnormal distribution of data was presented as median (interquartile range) and categorical data using number (percentage).

**Table 4:** Height SDS for patients by number of years treated.

Treatment years	Years treated						
	0 (*n* = 484)	1 (*n* = 292)	2 (*n* = 106)	3 (*n* = 44)	4 (*n* = 20)	5 (*n* = 9)	6 (*n* = 4)
0	−2.9 ± 0.8	−3.0 ± 1.0	−3.1 ± 1.2	−3.4 ± 1.5	−3.9 ± 1.8	−3.4 ± 1.5	−3.4 ± 1.0
1	—	−1.5 ± 0.9	−1.5 ± 0.9	−1.5 ± 0.8	−1.7 ± 0.7	−1.8 ± 0.6	−1.4 ± 0.2
2	—	—	−1.1 ± 0.8	−1.2 ± 0.7	−1.4 ± 0.6	−1.6 ± 0.4	−1.3 ± 0.2
3	—	—	—	−1.0 ± 0.9	−1.2 ± 0.9	−1.3 ± 0.8	−1.2 ± 0.3
4	—	—	—	—	−1.1 ± 0.8	−1.1 ± 0.7	−1.1 ± 0.3
5	—	—	—	—	—	−1.0 ± 0.7	−1.0 ± 0.2
6	—	—	—	—	—	—	−0.8 ± 0.3

Abbreviations: height SDS: height standard deviation scores.

## Data Availability

The data used to support the findings of this study are available from the corresponding author upon request.

## Supplementary Materials

Supplementary Materials Table S1: Height SDS of patients with GHD by number of years treated. Table S2: Height SDS of patients with ISS by number of years treated. Table S3: Height SDS of patients with FSS by number of years treated. Table S4: Height SDS of patients with hypothyroidism by number of years treated. Table S5: Height SDS of patients with SGA by number of years treated.

## References

[1] Maghnie M., Labarta J. I., Koledova E., Rohrer T. R. (2018). Short stature diagnosis and referral. *Frontiers in Endocrinology*.

[2] Wang Q., Liu D. Y., Yang L. Q., Liu Y., Chen X. J. (2015). The epidemic characteristics of short stature in school students. *Italian Journal of Pediatrics*.

[3] Allen D. B., Cuttler L. (2013). Clinical practice. Short stature in childhood--challenges and choices. *The New England Journal of Medicine*.

[4] Yue D., Miller M. R., Clarson C. L. (2019). Evaluation of referrals for short stature: a retrospective chart review. *Paediatrics & Child Health*.

[5] Aguirre G. A., De Ita J. R., de la Garza R. G., Castilla-Cortazar I. (2016). Insulin-like growth factor-1 deficiency and metabolic syndrome. *Journal of Translational Medicine*.

[6] Sommer R., Bullinger M., Chaplin J. (2017). Experiencing health-related quality of life in paediatric short stature - a cross-cultural analysis of statements from patients and parents. *Clinical Psychology & Psychotherapy*.

[7] Naiki Y., Horikawa R., Tanaka T. (2013). Assessment of psychosocial status among short-stature children with and without growth hormone therapy and their parents. *Clinical Pediatric Endocrinology*.

[8] Collett-Solberg P. F., Ambler G., Backeljauw P. F. (2019). Diagnosis, genetics, and therapy of short stature in children: a growth hormone research society international perspective. *Hormone Research in Pædiatrics*.

[9] Pfäffle R., Land C., Schönau E. (2018). Growth hormone treatment for short stature in the USA, Germany and France: 15 years of surveillance in the genetics and neuroendocrinology of short-stature international study (GeNeSIS). *Hormone Research in Pædiatrics*.

[10] Khadilkar V., Ekbote V., Kajale N., Khadilkar A., Chiplonkar S., Kinare A. (2013). Effect of one-year growth hormone therapy on body composition and cardio-metabolic risk in Indian children with growth hormone deficiency. *Endocrine Research*.

[11] Chen M., Gan D., Luo Y. (2018). Effect of recombinant human growth hormone therapy on blood lipid and carotid intima-media thickness in children with growth hormone deficiency. *Pediatric Research*.

[12] Gonzalez B. L., Viaud M., Beltrand J. (2019). Improved general and height-specific quality of life in children with short stature after one year on growth hormone. *The Journal of Clinical Endocrinology and Metabolism*.

[13] Wu S., Liu Q., Gu W., Ni S., Shi X., Zhu Z. (2018). A retrospective analysis of patients with short stature in the south of China between 2007 and 2015. *BioMed Research International*.

[14] Rogol A. D., Hayden G. F. (2014). Etiologies and early diagnosis of short stature and growth failure in children and adolescents. *The Journal of Pediatrics*.

[15] Li H., Ji C. Y., Zong X. N., Zhang Y. Q. (2009). Height and weight standardized growth charts for Chinese children and adolescents aged 0 to 18 years. *Chinese Journal Of Pediatrics*.

[16] Li H., Ji C. Y., Zong X. N., Zhang Y. Q. (2009). Body mass index growth curves for Chinese children and adolescents aged 0 to 18 years. *Chinese Journal Of Pediatrics*.

[17] Wright C. M., Ahmed L., Dunger D. B., Preece M. A., Cole T. J., Butler G. (2012). Can we characterise growth in puberty more accurately? Validation of a new Puberty Phase Specific (PPS) growth chart. *Archives of Disease in Childhood*.

[18] Diseases H. M. (2008). Guidelines for diagnosis and treatment of children with short stature. *Chinese Journal Of Pediatrics*.

[19] Isojima T., Shimatsu A., Yokoya S. (2012). Standardized centile curves and reference intervals of serum insulin-like growth factor-I (IGF-I) levels in a normal Japanese population using the LMS method. *Endocrine Journal*.

[20] Satoh M. (2015). Bone age: assessment methods and clinical applications. *Clinical Pediatric Endocrinology*.

[21] Liu S., Ban B., Pan H. (2017). The research of prevalence of short stature of children and adolescents aged from 6 to 16 years in Jining. *Chinese Journal of Diagnostics(Electronic Edition)*.

[22] Scherdel P., Dunkel L., van Dommelen P. (2016). Growth monitoring as an early detection tool: a systematic review. *The Lancet Diabetes & Endocrinology*.

[23] Saengkaew T., McNeil E., Jaruratanasirikul S. (2017). Etiologies of short stature in a pediatric endocrine clinic in Southern Thailand. *Journal of Pediatric Endocrinology and Metabolism*.

[24] Song K. C., Jin S. L., Kwon A. R. (2015). Etiologies and characteristics of children with chief complaint of short stature. *Annals of Pediatric Endocrinology & Metabolism*.

[25] Ranke M. B., Lindberg A., Tanaka T., Camacho-Hübner C., Dunger D. B., Geffner M. E. (2017). Baseline characteristics and gender differences in prepubertal children treated with growth hormone in Europe, USA, and Japan: 25 years’ KIGS® experience (1987-2012) and review. *Hormone Research in Pædiatrics*.

[26] Grimberg A., Huerta-Saenz L., Grundmeier R. (2015). Gender bias in U.S. pediatric growth hormone treatment. *Scientific Reports*.

[27] Polak M., Blair J. C., Kotnik P., Pournara E., Pedersen B. T., Rohrer T. R. (2017). Early growth hormone treatment start in childhood growth hormone deficiency improves near adult height: analysis from NordiNet International Outcome Study. *European Journal of Endocrinology*.

[28] Reiter E. O., Price D. A., Wilton P., Albertsson-Wikland K., Ranke M. B. (2006). Effect of growth hormone (GH) treatment on the near-final height of 1258 patients with idiopathic GH deficiency: analysis of a large international database. *The Journal of Clinical Endocrinology & Metabolism*.

[29] de Pedro S., Murillo M., Salinas I. (2016). Variability in adherence to rhGH treatment: Socioeconomic causes and effect on children's growth. *Growth Hormone & IGF Research*.

[30] Haverkamp F., Johansson L., Dumas H. (2008). Observations of nonadherence to recombinant human growth hormone therapy in clinical practice. *Clinical Therapeutics*.

[31] Ying Y. Q., Hou L., Liang Y., Wu W., Luo X. P. (2018). Efficacy and safety of recombinant human growth hormone in treating Chinese children with idiopathic short stature. *Growth Hormone & IGF Research*.

[32] Kemp S. F., Kuntze J., Attie K. M. (2005). Efficacy and safety results of long-term growth hormone treatment of idiopathic short stature. *The Journal of Clinical Endocrinology and Metabolism*.

