# Supplementary material for: A Retrospective Analysis of Patients with Short Stature in Eastern China between 2013 and 2019
Source: Biomed Res Int. 2021 Apr 20;2021:6640026. doi: 10.1155/2021/6640026 (PMC8081605; doi:10.1155/2021/6640026)
Supplement: Supplementary Materials — Table S1: Height SDS of patients with GHD by number of years treated. Table S2: Height SDS of patients with ISS by number of years treated. Table S3: Height SDS of patients with FSS by number of years treated. Table S4: Height SDS of patients with hypothyroidism by number of years treated. Table S5: Height SDS of patients with SGA by number of years treated. [file 6640026.f1.doc]

Table S1: Height SDS of patients with GHD by number of years treated.

| Treatment years |  | Years treated | | | | | |
| --- | --- | --- | --- | --- | --- | --- | --- |
|  | 0 (n=271) | 1 (n = 178) | 2 (n = 56) | 3 (n = 26) | 4 (n = 12) | 5 (n = 5) | 6 (n = 2) |
| 0 | -2.9 ± 0.8 | -3.0 ± 1.0 | -3.3 ± 1.3 | -3.7 ± 1.7 | -3.9 ± 1.8 | -3.4 ± 1.3 | -3.4 ± 1.0 |
| 1 | - | -1.4 ± 0.8 | -1.4 ± 0.9 | -1.5 ± 0.7 | -1.5 ± 0.6 | -1.8 ± 0.6 | -1.6 ± 0.0 |
| 2 | - | - | -1.1 ± 0.9 | -1.2 ± 0.7 | -1.2 ± 0.7 | -1.6 ± 0.4 | -1.2 ± 0.0 |
| 3 | - | - | - | -0.9 ± 0.8 | -0.8 ± 0.7 | -1.2 ± 0.9 | -1.1 ± 0.0 |
| 4 | - | - | - | - | -0.9 ± 0.9 | -1.1 ± 0.8 | -0.9 ± 0.0 |
| 5 | - | - | - | - | - | -0.9 ± 0.2 | -0.9 ± 0.0 |
| 6 | - | - | - | - | - | - | -0.8 ± 0.0 |

Abbreviations: Height SDS: height standard deviation scores; GHD: growth hormone defciency.

Table S2: Height SDS of patients with ISS by number of years treated.

| Treatment years |  | Years treated | | |
| --- | --- | --- | --- | --- |
|  | 0 (n=85) | 1 (n = 42) | 2 (n = 17) | 3 (n = 3) |
| 0 | -3.0 ± 0.9 | -2.9 ± 0.8 | -3.2 ± 1.2 | -3.2 ± 1.1 |
| 1 | - | -1.4 ± 1.0 | -1.3 ± 1.0 | -0.8 ± 0.9 |
| 2 | - | - | -1.0 ± 0.9 | -0.8 ± 0.7 |
| 3 | - | - | - | -0.6 ± 0.5 |
| 4 | - | - | - | - |
| 5 | - | - | - | - |
| 6 | - | - | - | - |

Abbreviations: Height SDS: height standard deviation scores; ISS: idiopathic short stature.

Table S3: Height SDS of patients with FSS by number of years treated.

| Treatment years |  | Years treated | | | | |
| --- | --- | --- | --- | --- | --- | --- |
|  | 0 (n=63) | 1 (n = 39) | 2 (n = 17) | 3 (n = 6) | 4 (n = 2) | 5 (n =1) |
| 0 | -2.7 ± 0.6 | -2.7 ± 0.7 | -2.9 ± 0.9 | -2.3 ± 0.2 | -2.4 ± 0.3 | -2.7 ± 0.0 |
| 1 | - | -1.5 ± 1.1 | -1.5 ± 1.0 | -1.2 ± 0.8 | -2.1 ± 0.8 | -2.4 ± 0.0 |
| 2 | - | - | -1.1 ± 0.8 | -1.0 ± 0.9 | -2.1 ± 0.1 | -2.0 ± 0.0 |
| 3 | - | - | - | -0.8 ± 0.6 | -1.9 ±0.0 | -1.9 ± 0.0 |
| 4 | - | - | - | - | -1.4 ± 0.0 | -1.5 ± 0.0 |
| 5 | - | - | - | - | - | -1.3 ± 0.0 |
| 6 | - | - | - | - | - | - |

Abbreviations: Height SDS: height standard deviation scores; FSS: familial short stature.

Table S4: Height SDS of patients with hypothyroidism by number of years treated.

| Treatment years |  | Years treated | | | | | |
| --- | --- | --- | --- | --- | --- | --- | --- |
|  | 0 (n=26) | 1 (n = 18) | 2 (n = 9) | 3 (n = 4) | 4 (n = 4) | 5 (n = 2) | 6 (n = 1) |
| 0 | -2.9 ± 0.8 | -2.6 ± 0.4 | -2.5 ± 0.4 | -2.5 ± 0.2 | -2.5 ± 0.2 | -2.6 ± 0.2 | -2.4 ± 0.0 |
| 1 | - | -1.7 ± 0.8 | -1.7 ± 0.5 | -2.0 ± 0.7 | -2.1 ± 0.5 | -1.9 ± 0.4 | -1.6 ± 0.0 |
| 2 | - | - | -1.4 ± 0.3 | -1.6 ± 0.4 | -1.6 ± 0.4 | -1.6 ± 0.0 | -1.6 ± 0.0 |
| 3 | - | - | - | -1.5 ± 1.1 | -1.7 ± 1.3 | -1.5 ± 0.0 | -1.5 ± 0.0 |
| 4 | - | - | - | - | -1.3 ± 1.0 | -1.4 ± 0.9 | -0.9 ± 0.0 |
| 5 | - | - | - | - | - | -1.1 ± 0.2 | -0.8 ± 0.0 |
| 6 | - | - | - | - | - | - | -0.4 ± 0.0 |

Abbreviations: Height SDS: height standard deviation scores.

Table S5: Height SDS of patients with SGA by number of years treated.

| Treatment years |  | Years treated | | | | | |
| --- | --- | --- | --- | --- | --- | --- | --- |
|  | 0 (n=15) | 1 (n = 8) | 2 (n = 5) | 3 (n = 3) | 4 (n = 2) | 5 (n = 1) | 6 (n = 1) |
| 0 | -3.1 ± 1.1 | -3.3 ± 1.3 | -2.7 ± 0.6 | -3.0 ± 1.0 | -2.5 ± 0.1 | -2.4 ± 0.0 | -2.4 ± 0.0 |
| 1 | - | -2.0 ± 1.0 | -1.5 ± 0.7 | -1.7 ± 0.9 | -1.7 ± 1.1 | -1.3 ± 0.0 | -1.3 ± 0.0 |
| 2 | - | - | -1.3 ± 0.7 | -1.4 ± 0.8 | -1.4 ± 0.8 | -1.2 ± 0.0 | -1.2 ± 0.0 |
| 3 | - | - | - | -1.3 ± 0.8 | -1.3 ± 0.7 | -1.2 ± 0.0 | -1.2 ± 0.0 |
| 4 | - | - | - | - | -1.2 ± 0.8 | -1.1 ± 0.0 | -1.1 ± 0.0 |
| 5 | - | - | - | - | - | -1.1 ± 0.0 | -1.1 ± 0.0 |
| 6 | - | - | - | - | - | - | -0.8 ± 0.0 |

Abbreviations: Height SDS: height standard deviation scores; SGA: small for gestational age.
